# A Case of Trisomy 9 Mosaicism Diagnosed Following Detection of Placental Trisomy 9

**DOI:** 10.7759/cureus.85532

**Published:** 2025-06-07

**Authors:** Yuri Hasegawa, Shoko Miura, Koh Nagata, Ai Nagata, Kiyonori Miura

**Affiliations:** 1 Obstetrics and Gynecology, Nagasaki University Graduate School of Biomedical Sciences, Nagasaki, JPN

**Keywords:** confined placental mosaicism, fetal growth restriction, placental chromosome analysis, placental trisomy 9, trisomy 9 mosaicism, true fetal mosaicism

## Abstract

We report a case in which fetal growth restriction was observed during pregnancy. A placental chromosomal analysis was performed to investigate the cause of the fetal growth restriction and it showed trisomy 9. Prompt chromosomal testing of the neonate led to the diagnosis of trisomy 9 mosaicism. Although the neonate was small for gestational age and had mild respiratory distress and feeding difficulties, her clinical symptoms were minimal. Trisomy 9 mosaicism is extremely rare and shows considerable variability in its clinical presentation. This case report is important because trisomy 9 was detected by a placental chromosome analysis that was conducted to investigate the cause of fetal growth restriction. Additionally, a newborn with mild clinical findings was unexpectedly diagnosed with trisomy 9 mosaicism.

## Introduction

Mosaic trisomy 9 is a rare chromosomal abnormality presenting with diverse clinical features [1], including intellectual disability, growth retardation, craniofacial anomalies, congenital heart defects, urogenital anomalies, skeletal abnormalities, and central nervous system malformations. The exact number is not specified; over 100 cases have been reported [2]. However, the true incidence remains unclear. A small number of cases with minimal or no structural anomalies have also been described, suggesting that some affected individuals may have a more favorable prognosis [3,4]. Due to its rarity and phenotypic diversity, prenatal diagnosis and prognostic counseling for trisomy 9 mosaicism remain challenging. Fetal growth restriction (FGR) is commonly observed during the fetal period [2]. Confined placental mosaicism (CPM) is known to cause FGR. CPM is defined as the presence of chromosomally abnormal cell lines confined to the placenta, while the fetal karyotype remains normal [5,6]. CPM may involve either exclusively abnormal cell lines or a mixture of normal and abnormal cell lines within the placental tissue. A recent meta-analysis reported an elevenfold increased risk of FGR in cases with CPM compared to those without [7]. Herein, we report a case in which FGR was observed during pregnancy, and placental chromosomal analysis revealed full trisomy 9, leading to the diagnosis of neonatal trisomy 9 mosaicism. This case is reported with the consent of the parents.

## Case presentation

A 34-year-old woman, gravida 2 para 1, conceived naturally and underwent regular prenatal checkups at a local clinic. Her estimated due date was determined based on her last menstrual period, which was consistent with crown-rump length at 9 weeks' gestation. She transferred to our hospital at 14 weeks' gestation following relocation. At 20 weeks, fetal screening by transabdominal ultrasound showed no abnormalities. From 28 weeks, fetal growth lagged behind by -1.5 SD, without evidence of abnormal Doppler findings. TORCH syndrome was considered a possible cause of FGR, and blood tests were performed, but all results were negative.

At 34 weeks, decreased amniotic fluid and variable decelerations associated with uterine contractions were observed on cardiotocography (CTG). The patient was hospitalized for management due to suspected FGR and fetal compromise. Estimated fetal weight was 1,674g (-1.9 SD), and maximum vertical pocket of amniotic fluid was 3.0 cm. Umbilical artery Doppler showed no absent or reversed end-diastolic flow. Occasional CTG abnormalities were noted during hospitalization.

**Figure 1:**
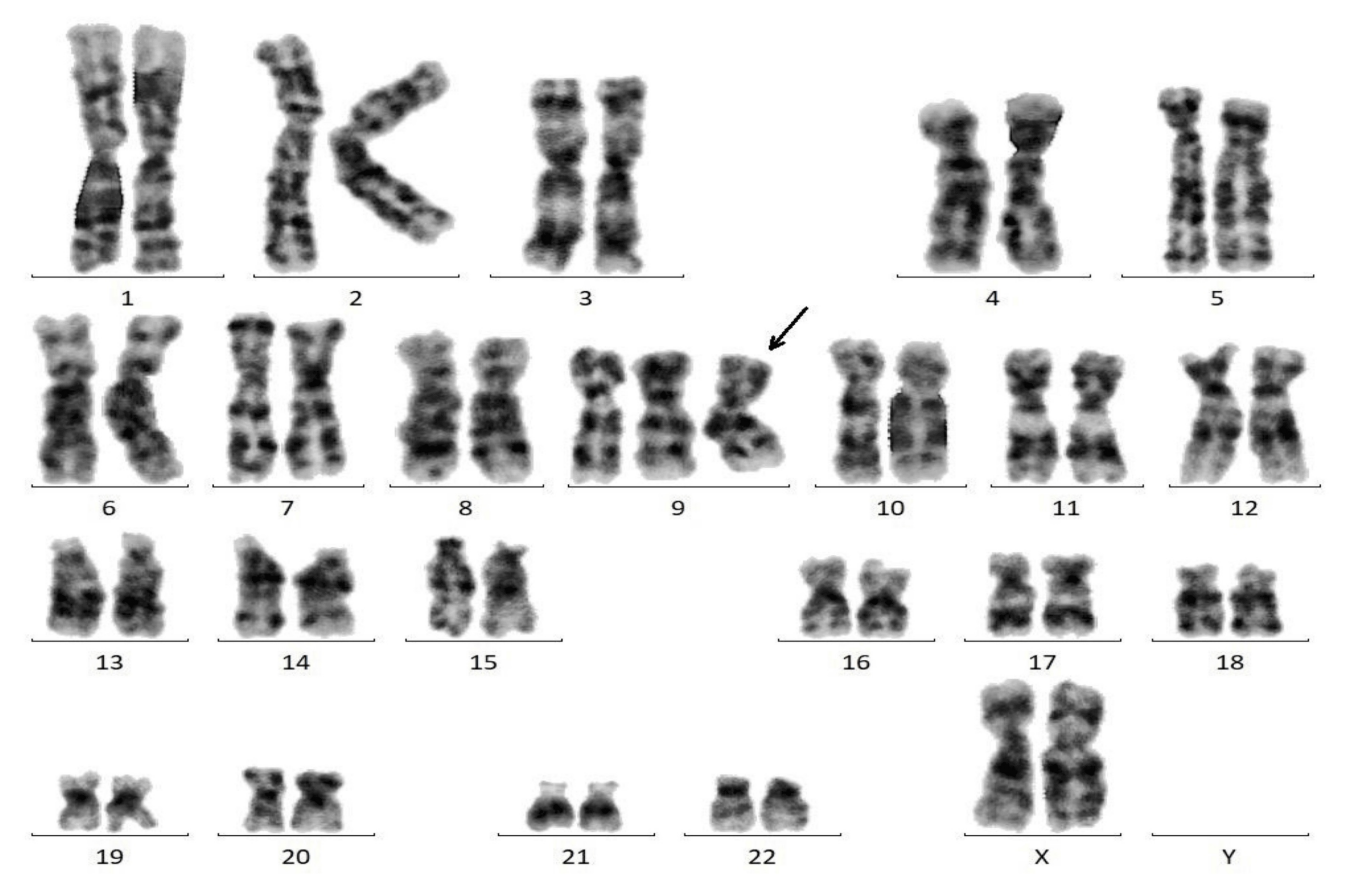
Chromosomal analysis of the placenta. All 50 cells showed 47,XX,+9 (black arrow).

Labor was induced at 37 weeks 0 days, and emergency cesarean section was performed at 37 weeks 1 day due to repeated late and severe variable decelerations. A female neonate weighing 1,754g (0.2 percentile) was delivered, with Apgar scores of 8 and 9 at 1 and 5 minutes, respectively, and an umbilical artery pH of 7.33. The placenta weighed 246g and showed no abnormalities in the umbilical cord or insertion site. The placenta was collected immediately after delivery. CPM was suspected as a cause of small for gestational age (SGA), and chromosomal analysis of the placenta was performed with parental consent. Placental tissue samples were collected and submitted to Fetal Life Science Center, Ltd., located in Nagoya, Japan, for chromosomal analysis. The samples were processed and analyzed using G-banding (Giemsa staining), a standard cytogenetic method for detecting chromosomal abnormalities. A total of 50 metaphase cells were examined to enhance the sensitivity for detecting mosaicism and structural variations. G-banding analysis of 50 metaphase cells revealed a karyotype of 47,XX,+9, indicating trisomy 9 (Figure 1).

Neonatal findings

The neonate was admitted to the neonatal intensive care unit due to SGA and low birth weight. No external malformations or complications were initially noted. No facial features characteristic of trisomy 9 mosaicism were observed. Urinary CMV-DNA was negative. She required nasal continuous positive airway pressure therapy for seven days due to neonatal respiratory distress. Feeding difficulties led to an upper gastrointestinal contrast study, which diagnosed laryngomalacia. Fiberoptic laryngoscopy revealed gastroesophageal reflux.

**Figure 2:**
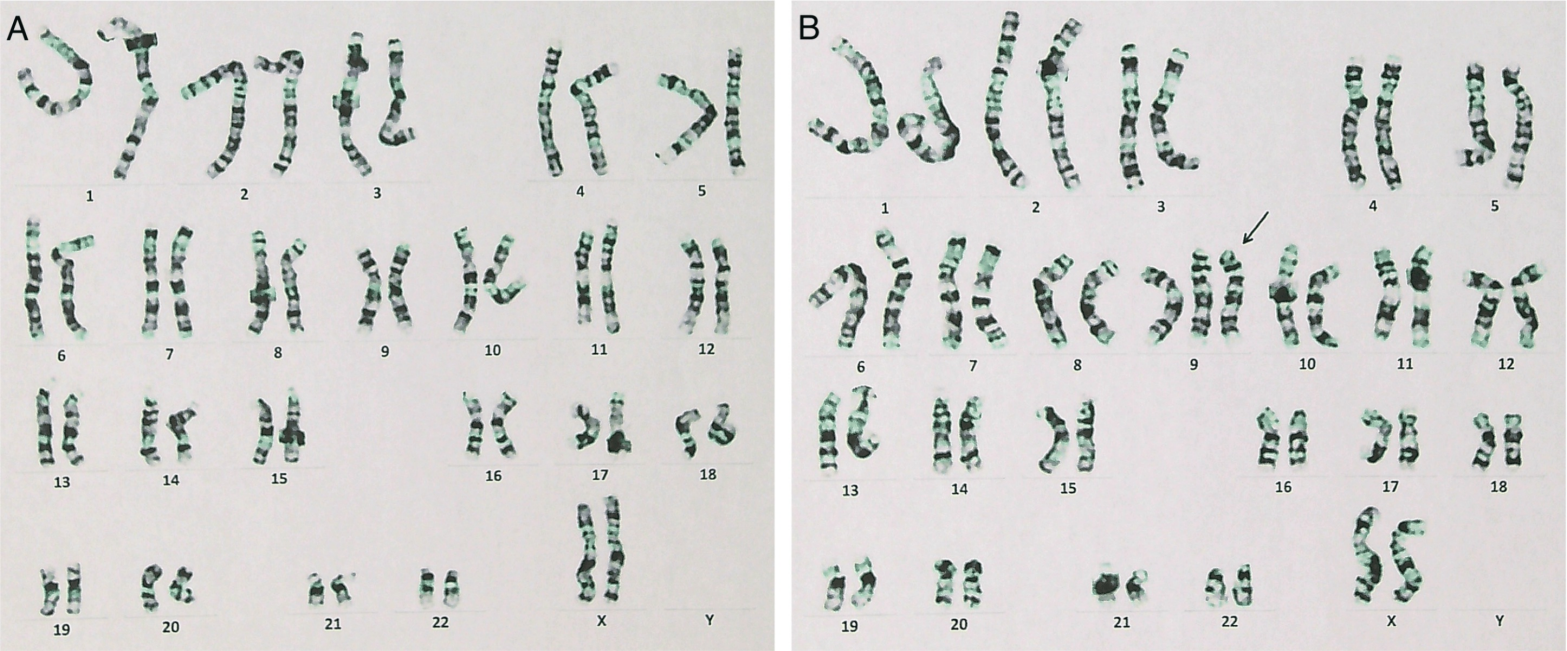
Chromosomal analysis of the neonatal blood. The results of the neonatal chromosome test were 47,XX,+9[8 cells]/46,XX[42 cells], indicating a mosaic rate of 16%. A: normal karyotype, B: trisomy 9 (black arrow)

Following the detection of trisomy 9 in the placenta, the parents consented to neonatal chromosomal testing. Peripheral blood chromosomal analysis was performed at LSI Medience using standard G-banding. Lymphocytes were cultured for 72 hours, arrested in metaphase, and stained with Giemsa. At least 20 metaphase cells were analyzed according to ISCN guidelines to detect numerical and structural chromosomal abnormalities. The result of neonatal chromosomal testing revealed 47,XX,+9[8 cells]/46,XX[42 cells], indicating a mosaic rate of 16% (Figure 2A; normal karyotype, Figure 2B; trisomy 9). Home enteral feeding was necessary due to poor oral intake, and the infant was discharged on day 52.

Genetic counseling

Genetic counseling was provided on day 23 postpartum following the placental chromosomal findings. The possibility that the neonate had either full trisomy 9 or trisomy 9 mosaicism was explained. Since no external malformations were evident and live-born cases of full trisomy 9 are extremely rare, full trisomy 9 was considered unlikely. The possibility of trisomy 9 mosaicism remained.

Following neonatal chromosomal testing confirming mosaicism, further counseling was conducted. Clinical manifestations of trisomy 9 mosaicism range from mild to severe. At present, no life-threatening congenital heart defects, severe respiratory disorders, or central nervous system abnormalities were identified. Future management would focus on monitoring developmental milestones and emerging symptoms. The mother, tearful, shared that she had researched the condition online and was concerned by reports of poor prognosis. Information about family support groups for trisomy 9 mosaicism was provided to alleviate parental anxiety.

A literature review of clinical characteristics of cases of trisomy 9 mosaicism without apparent morphological anomalies

As in this case, mosaicism with trisomy 9 without morphological abnormalities is extremely rare. Table 1 shows a review of mosaicism of trisomy 9 without morphological abnormalities. These three cases represent rare phenotypic variants of trisomy 9 mosaicism in which no apparent structural anomalies were detected. In particular, the case described by Hai et al. demonstrates that partial trisomy 9 mosaicism may be compatible with normal physical and cognitive development as well as successful reproduction [3].

**Table 1:** Clinical characteristics of four cases of trisomy 9 mosaicism without apparent morphological anomalies. aCGH: Array comparative genomic hybridization; NIPT: Non-invasive prenatal testing; CMA: Chromosomal microarray analysis; FISH: Fluorescence in situ hybridization.

Case	Reference	Diagnostic Method	Mosaic Ratio	Presence of Morphological Anomalies	Perinatal prognosis
Case 1	Hai et al. [3]	aCGH	Not reported	None (only mild physical findings)	Normal mental/motor, healthy child
Case 2	Ma et al. [4]	NIPT, karyotyping, CMA, FISH	42% in amniocytes; 22.4% (CMA) and 34% (FISH) in cord blood	None (no abnormal findings on ultrasound)	Artificial abortion
Case 3	Ma et al. [4]	NIPT, karyotyping, CMA, FISH	50% in amniocytes; 76% in placenta	None (no abnormal findings on ultrasound)	Artificial abortion
Case 4	Our case	Karyotyping	16％ in the blood of the neonate; 100% in placenta	None (only mild physical findings)	Breastfeeding difficulties

Furthermore, the two cases reported by Ma et al. [4] show tissue-specific variability in mosaic ratios across different fetal organs, suggesting that phenotypic expression may depend on the extent and distribution of mosaicism. These findings contribute to our understanding of the clinical heterogeneity of trisomy 9 mosaicism and emphasize the importance of individualized genetic counseling and prognostication.

## Discussion

**Figure 3:**
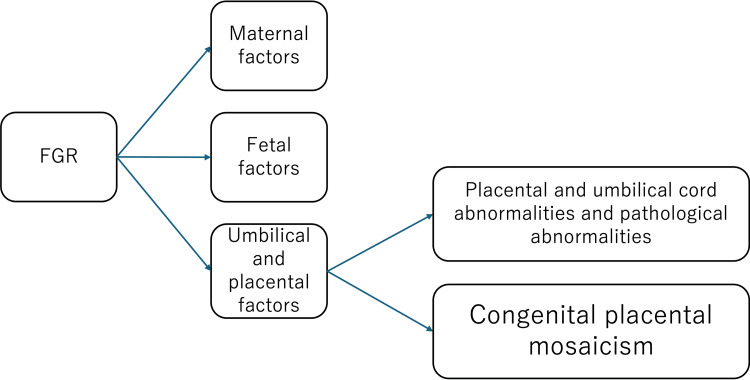
The protocol for investigating the causes of FGR at our hospital. CPM is suspected when no maternal or fetal factors are identified and no morphological or pathological abnormalities are found in the placenta or umbilical cord. FGR: Fetal growth restriction.

This case represents a rare instance where full trisomy 9 was identified via placental chromosomal testing during FGR evaluation, leading to neonatal diagnosis of trisomy 9 mosaicism. Figure 3 shows the protocol for investigating the causes of FGR at our hospital. If no maternal or fetal factors are identified and no morphological or pathological abnormalities are found in the placenta or umbilical cord, CPM is suspected, and placental chromosome testing is performed.

Common features reported in trisomy 9 mosaicism include craniofacial anomalies, cardiac defects, feeding and respiratory difficulties, cryptorchidism, hip dysplasia, seizures, and developmental delay [2,7]. However, in this case, no typical congenital anomalies were observed prenatally or neonatally, except for FGR and mild neonatal complications. Therefore, if no abnormalities had been detected in the placental chromosome analysis, the diagnosis of neonatal 9-trisomy mosaicism might have been delayed. This case report is a very important finding that highlights the importance of placental chromosome analysis in FGR.

Clinical features of trisomy 9 mosaicism and genetic counseling for parents

The neonate's mosaic rate was 16%, comparable to previously reported averages (range: 4-80%, mean: 31%, median: 20%) [2]. Without placental chromosomal testing, neonatal chromosomal testing would not have been initially considered. Placental chromosomal analysis facilitated early diagnosis and guided appropriate follow-up.

Only one previous report [7] has described a similar case where placental trisomy 9 was associated with neonatal trisomy 9 mosaicism. In that case, despite a low mosaic rate (3.3%), the neonate exhibited severe clinical manifestations. Trisomy 9 mosaicism exhibits wide clinical variability [2,8], and the mosaic rate does not reliably predict severity, an important point to convey during genetic counseling. Although no morphological abnormalities were observed in the neonate in this case, the long-term motor and neurodevelopmental outcomes remain uncertain, and careful follow-up will be necessary.

CPM should always be considered in the differential diagnosis of FGR [9,10]. In the present case, chromosomal testing of the neonate was not initially planned, given the relatively mild clinical symptoms. However, identification of trisomy 9 through placental chromosomal analysis, conducted to investigate the cause of FGR, prompted neonatal chromosomal testing. Future follow-up for the child will involve careful monitoring for developmental delay, epilepsy, and ophthalmological disorders, which are characteristic of trisomy 9 mosaicism. Thus, placental chromosomal testing is considered an important examination that can contribute to understanding the child's prognosis and guide subsequent follow-up.

Mechanism of occurrence in this case

In this case, full trisomy 9 was detected in the placental tissue, while the neonate exhibited mosaicism for trisomy 9. This discordance between the chromosomal constitution of the placenta and the fetus fulfills the diagnostic criteria for true fetal mosaicism. Two major mechanisms have been proposed to explain the formation of such mosaicism.

The first mechanism involves post-zygotic mitotic errors occurring during early embryonic development. In this scenario, a chromosomally normal zygote (46,XX or 46,XY) undergoes mitotic nondisjunction or chromosome loss during cleavage-stage divisions, resulting in the generation of a mixed cell population, some with the normal diploid karyotype and others with trisomy. If this mitotic error affects cells of the inner cell mass, it can give rise to a fetus with chromosomal mosaicism [5,6].

The second mechanism involves meiotic nondisjunction, leading to the formation of a trisomic zygote (47,XX,+9 or 47,XY,+9) at the time of fertilization. Subsequently, during early post-zygotic mitoses, one of the three copies of chromosome 9 may be lost in a subset of cells, a process known as trisomy rescue. This results in a mosaic embryo composed of both trisomic and corrected diploid cells [6,11].

In the present case, it is challenging to definitively identify which mechanism was responsible for the observed mosaicism. However, the presence of uniform full trisomy 9 in the placental tissue suggests that the chromosomal abnormality was present from the earliest stages of development. If the embryo had originally been chromosomally normal, one would expect to observe a mixture of normal and abnormal cells in the placenta, rather than a uniform trisomic pattern. Therefore, the findings are more consistent with an initial meiotic nondisjunction event, resulting in a trisomic zygote, followed by post-zygotic correction in the fetal lineage. This interpretation supports the notion that the mosaicism in the fetus arose from trisomy rescue, rather than a de novo mitotic error in an originally euploid embryo.

## Conclusions

Placental chromosome analysis is an important test for identifying the cause of FGR. This case is a very rare example in which placental trisomy 9 led to the diagnosis of trisomy 9 mosaicism in a newborn. Placental chromosome testing can sometimes provide essential insights for the prognosis and follow-up of neonates. While placental testing was pivotal here, its utility depends on the clinical context.
